# Perceived Self-Efficacy, Gender Roles, and Cultural Identity As Determinants of Vascular and Neurovascular Health in Métis People in Canada

**DOI:** 10.1016/j.cjco.2026.05.015

**Published:** 2026-06-04

**Authors:** Shara R. Johnson, Samantha Moore, Phil Chilibeck, Gavin Selinger, Adam McInnes, Heather J.A. Foulds

**Affiliations:** a College of Kinesiology, University of Saskatchewan, Saskatoon, Saskatchewan, Canada; b Member of Saskatoon Métis Local 126, Citizen of Métis Nation-Saskatchewan, Saskatoon, Saskatchewan, Canada; c Division of Biomedical Engineering, College of Engineering, University of Saskatchewan, Saskatoon, Saskatchewan, Canada

**Keywords:** Indigenous peoples, cardiovascular health, social determinants

## Abstract

**Background:**

Métis people, a distinct Indigenous group in Canada, have unique culture, identity, and experiences, although they have a shared history of colonization with other Indigenous groups. Although they are actively reclaiming and revitalizing their culture, identity, and community connections, Métis people remain disproportionally burdened by chronic illnesses, including cardiovascular diseases. This study examined relationships of psychosocial factors—self-efficacy, cultural identity affiliation, and gender role responsibilities—with established cardiovascular risk indicators in Métis people.

**Methods:**

Métis adults (N = 72, 67% female, mean age = 39 ± 16 years) were invited to complete questionnaires assessing self-efficacy, cultural identity, and gender roles responsibility. Vascular and neurovascular indicators were assessed using finger photoplethysmography and infrared photoelectric sensors with a 3-lead electrocardiograph.

**Results:**

Self-efficacy (β = –0.34, *P* < 0.01) and Métis identity (β = 0.29, *P* = 0.01) were associated with lower-body peripheral pulse wave velocity, reflecting decreased and increased arterial stiffness, respectively. Indoor housework responsibility moderated the relationship between self-efficacy and baroceptor sensitivity (β = 0.28, *P* = 0.03). Self-efficacy moderated by outdoor housework responsibility was associated with high frequency heart rate variability, indicating higher parasympathetic activation. Specifically, Métis persons with high levels of self-efficacy and responsibility for outdoor housework had worse heart rate variability than did persons with a high level of self-efficacy and a lower level of outdoor housework responsibility.

**Conclusions:**

Self-efficacy, housework roles, and cultural identity affiliation contribute significantly to some vascular and neurovascular health parameters in Métis people. There is an opportunity to enhance vascular and neurovascular health by developing community-driven strength-based approaches, grounded in Métis peoples’ resilience, self-determination, and cultural identity.

## Résumé

**Contexte:**

Les Métis, un groupe autochtone distinct au Canada, possèdent une culture, une identité et des expériences qui leur sont propres, tout en partageant avec les autres groupes autochtones une histoire commune marquée par la colonisation. Bien qu’ils s’emploient activement à se réapproprier et à revitaliser leur culture, leur identité et leurs liens communautaires, les Métis restent touchés de manière disproportionnée par des maladies chroniques, notamment les maladies cardiovasculaires. Cette étude a examiné les relations entre certains facteurs psychosociaux — l’auto-efficacité, l’appartenance à une identité culturelle et les responsabilités liées aux rôles de genre — et des indicateurs de risque cardiovasculaire établis chez les Métis.

**Méthodologie:**

Des adultes métis (n = 72, 67 % de femmes, âge moyen = 39 ± 16 ans) ont été invités à remplir des questionnaires évaluant l’auto-efficacité, l’identité culturelle et les responsabilités liées aux rôles du genre. Les indicateurs vasculaires et neurovasculaires ont été évalués à l’aide d’une photopléthysmographie digitale et de capteurs photoélectriques infrarouges, associés à un électrocardiographe à trois dérivations.

**Résultats:**

L’auto-efficacité (β = -0,34, *p* < 0,01) et l’identité métisse (β = 0,29, *p* = 0,01) étaient associées à une vitesse d’onde de pouls périphérique plus faible dans le bas du corps, reflétant respectivement une diminution et une augmentation de la rigidité artérielle. La responsabilité des tâches ménagères d’intérieur modérait la relation entre l’auto-efficacité et la sensibilité des barorécepteurs (β = 0,28, *p* = 0,03). L’auto-efficacité, modulée par la responsabilité des tâches ménagères d’extérieur, était associée à une variabilité de la fréquence cardiaque en haute fréquence, indiquant une activation parasympathique plus élevée. Plus précisément, les personnes métisses présentant une auto-efficacité élevée et une forte responsabilité dans les tâches ménagères extérieures affichaient une moins bonne variabilité de la fréquence cardiaque que celles présentant une auto-efficacité élevée et une responsabilité moindre pour ces tâches.

**Conclusion:**

L’auto-efficacité, les rôles liés aux tâches domestiques et l’appartenance à une identité culturelle contribuent de manière significative à certains paramètres de santé vasculaire et neurovasculaire chez les Métis. Il est possible d’améliorer la santé vasculaire et neurovasculaire en développant des approches communautaires fondées sur les points forts, ancrées dans la résilience, l’autodétermination et l’identité culturelle des peuples métis.

Métis people, one of the constitutionally recognized Indigenous groups in Canada, have unique culture, identity, and experiences, though a shared history of colonization with other Indigenous nations.^1^^,^^2^ These experiences contribute to unique health outcomes, including disproportionate burdens of chronic diseases, such as cardiovascular (CV) diseases (CVDs), compared to non-Indigenous populations.^3^, ^4^, ^5^ CVD, a leading cause of death, also affects men and women differently, with higher mortality among women linked to psychosocial and behavioural factors, such as caregiving responsibilities, stress, depression, and physical inactivity.^6^, ^7^, ^8^

CV health disparities are influenced by broader psychosocial determinants, including cultural identity, social connections, and stress.^9^ Cultural identity characterized by group belonging and ties, and sense of self,^10^ can shape health behaviours, coping processes, and stress responses through neurophysiological pathways.^11^^,^^12^ Among Métis people, self-efficacy and cultural connectedness may serve as key protective factor for health, supporting resilience, self-determination, and overall well-being.^13^, ^14^, ^15^ At the same time, stress associated with psychosocial factors, including gendered household responsibilities and identity experiences, may influence CV functioning through activation of physiological stress pathways.^12^^,^^16^^,^^17^

Vascular and neurovascular measures, such as pulse wave velocity (PWV), heart rate variability (HRV), and baroreceptor sensitivity (BRS), are important indicators of CV risks and autonomic nervous system functioning^.^^18^^,^^19^ High PWV reflects increased arterial stiffness and elevated CVD risks,^20^^,^^21^ whereas lower HRV and BRS indicate impaired CV regulatory capacity.^22^ Psychosocial stressors may negatively influence these indicators through mechanisms such as inflammation, autonomic dysregulation, and endothelial dysfunction.^23^

Despite the growing recognition of the importance of psychosocial and cultural determinants of health, the relationships of self-efficacy, cultural identity, and gender roles with Métis CV health are relatively unknown. This study examined relationships of self-efficacy, Métis identity affiliation and gender role responsibilities with vascular and neurovascular health indicators in Métis people. The study also explored whether gender role responsibilities moderated the relationship between self-efficacy and vascular and neurovascular functioning. This aspect is important because self-efficacy reflects capacity to manage challenges, and gendered role responsibilities may influence stress exposure, workload burden, autonomy, and daily physical demands, potentially shaping how self-efficacy relates to CV functioning. Specifically, the research questions were as follows: (i) What is the nature of the relationship, if any, between self-efficacy, Métis identity affiliation, and gender roles (indoor and outdoor housework responsibility), and vascular and neurovascular health in Métis people; and (ii) Do relationships between self-efficacy and vascular and neurovascular health among Métis people differ based on gender role responsibilities?

## Methods

This observational study was done in Saskatchewan, Canada, homeland of Métis people, in collaboration with Saskatoon Métis Local 126. Study methods and results are reported following the **St**rengthening the **R**eporting of **Ob**servational Studies in **E**pidemiology (STROBE) statement for cross-sectional studies. Guided by the principles for Indigenous data governance, including collective benefit, authority to control, responsibility, and ethics, Métis values and cultural practices were integrated throughout the study. A community advisory committee (CAC), comprised of an elder and community representatives, provided direction on study design, recruitment, assessment tools, data use, and knowledge mobilization, ensuring Métis authority and shared governance.

Ethics approval was obtained from Saskatoon Métis Local 126 and the University of Saskatchewan Biomedical Research Ethics Board, reinforcing community governance and oversight.^24^ Convenience sampling was used to invite Métis adults living in Saskatchewan, through outlets including community networks, organizational partnerships, social media postings, and word of mouth. Additionally, participants were encouraged to share study information within their own networks, incorporating a snowballing approach to support recruitment. Saskatchewan has one of the highest proportions of Métis people, who comprise approximately 6% of the population.^1^^,^^25^^,^^26^ Data gathering occurred in person between February 2023 and August 2025 at University of Saskatchewan located in Saskatoon. Eligibility criteria included being at least 18 years of age, self-identification as Métis, and non-pregnancy at the time of assessment.

### Psychosocial assessments

Sociodemographic and health determinants information was gathered using questionnaires, including the New General Self-Efficacy Scale^27^; gender role information was gathered using the Family Roles indoor and outdoor housework subscales from the General Social Survey-Family^28^; and Métis identity affiliation was gathered using a Métis-specific scale codeveloped with the CAC, drawn from findings from an early phase of the larger project.^15^ Given the historical and ongoing impacts of colonization, which disrupted cultural continuity and led many Métis families to conceal their identity as a means of survival,^2^ the Métis identity affiliation scale was used to capture variation in participants’ engagement, discrimination experience, and sense of belonging to Métis culture and identity, rather than identity status itself. Sample questions included the following: (i) When you were growing up, did you know or acknowledge your Métis identity; and (ii) Do you engage in Métis social events, such as kitchen parties, kitchen dances, Métis dancing, or storytelling? Responses are scored from 1 (strongly disagree) to 5 (strongly agree) and were totaled, with higher scores indicating stronger affiliation with Métis identity. Reliability analyses showed acceptable to strong internal consistency across all scales (Cronbach’s α = 0.67-0.95).

### Clinical assessments

Anthropometric measures, systolic blood pressure (SBP), diastolic blood pressure (DBP), and heart rate (HR) were assessed using standardized protocols consistent with Canadian Society for Exercise Physiology protocols.^29^ Central and peripheral PWV vascular function measures were determined from the distance between 2 arterial sites divided by the pulse transit times between the 2 sites, including carotid to femoral (central PWV), carotid to finger (upper-body peripheral PWV), and femoral to toe (lower-body peripheral PWV).^20^^,^^30^Pulse transit times were measured using finger photoplethysmography (Finapress, Ohmeda, Englewood, CO) and infrared photoelectric sensors (ADInstruments, Colorado Springs, CO), and pulse-wave contours at the carotid, femoral, and toe arteries, with simultaneous 3-lead electrocardiograph. After 5 minutes of supine rest,^31^ 30 consecutive beats were recorded (Power Lab 8/35 and Lab Chart 8 software, ADInstruments). Neurovascular measures, HRV, and BRS were obtained simultaneously with PWV, drawn from 5 minutes of 3-lead electrocardiogram nodes and beat-to-beat finger arterial blood pressure (BP) recordings. Analyses using the BRS 6.3.0 software program (Nevrokard, Izola, Slovenia) assessed BRS, and HRV through spontaneous BP and HR fluctuations, including sequence and spectral analysis, and time and frequency domains.^32^

### Statistical analyses

Analysis using SPSS, version 28.0.1.0 statistical software suite (IBM, Armonk, NY) included determining means and standard deviations (SDs) for continuous variables; percentages for categorical variables were obtained for demographic, predictor, and outcome variables. Independent sample *t* tests examined sex differences in means. Pearsons’s *r* correlation examined intercorrelations among predictor and outcome variables. Hierarchical linear regression evaluated relationships of Métis identity, self-efficacy, and gender role responsibilities (indoor housework and outdoor housework) with vascular and neurovascular measures. Indoor and outdoor housework responsibilities were examined separately because they represent distinct socially patterned gender roles that may differentially influence stress, workload, and vascular and neurovascular health relationships.^33^ Regression models were built in 3 sequential steps based on theoretical, empirical, and community-informed evidence of factors influencing vascular and neurovascular health. Step 1 included established physiological covariates age, sex, SBP, and HR, which were identified a priori. Step 2 added the primary independent variables of interest, self-efficacy, housework responsibility, and Metis identity affiliation, to examine their independent associations with vascular and neurovascular outcomes beyond the covariates. Step 3 included the interaction term self-efficacy x housework responsibility to determine whether the relationship between self-efficacy and vascular and neurovascular health differed according to levels of gender role responsibility. Simple slopes scatterplots were used to analyze significant interaction effects. For the simple slopes examination, gender role responsibilities (indoor and outdoor housework) was categorized into the following 3 groups using SD cut-points: low (< −1 SD); shared (≥ −1 SD and ≤ 1 SD); and high (> 1 SD).

Using G∗Power sample size analysis and Cohen (1988) statistical power guidelines, a sample size of 68 was calculated to be sufficient to detect moderate effect sizes (f^2^ = 0.15) in multiple linear regression models with an alpha level of 0.05, power of 0.80, and up to 6 predictor variables and covariates.^34^^,^^35^ To avoid overfitting the regression models and maintain statistical power, predictors were grouped into 2 blocks, as follows: (i) self-efficacy, outdoor housework responsibility, and Métis identity; and (ii) self-efficacy, indoor housework responsibility, and Métis identity. Missing data points were handled using mean substitution, as the level of missingness was within commonly accepted thresholds and did not require a complex substitution approach. Assumptions of linearity, normality, multicollinearity, and homoscedasticity were met. Statistical significance was set at *P* < 0.05.

## Results

Demographic information and descriptive statistics for predictors and outcome variables are described in Table 1. Results from the *t* test (Table 1) indicated that the means of outdoor work differed significantly by sex, with male individuals having higher levels of outdoor housework responsibility than female individuals, which justified including sex as a covariate in the models. HR was higher for female individuals. No sex difference was present in the means for indoor housework responsibility, self-efficacy, Métis identity, or any of the vascular or neurovascular measures. Bivariate correlations (Supplemental Table S1) indicated significant correlations of vascular and neurovascular variables with age, SBP, and HR, justifying their inclusion as covariates in the models. Indoor and outdoor housework responsibility were significantly correlated, justifying separate blocks to minimize collinearity. Self-efficacy and Métis identity were correlated with lower-body peripheral (lpPWV).

**Table 1:** Demographic characteristics, by sex and overall participant group, of Métis people living in Saskatchewan

Characteristic	Female participants (n = 48)	Male participants (n = 24)	Overall participants (n = 72)	Missing cases	*P*∗
**Gender identity**					
Woman	46 (96)	0	46 (64)		
Man	0	24 (100)	24 (33)		
Gender-fluid	2 (4)	0	2 (3)		
Age, Y	40 ± 18	38 ± 13	39 ± 16		
**Education**					
High school	8 (16)	5 (21)	13 (18)		
Vocational school	18 (38)	8 (33)	26 (37)		
College/university	12 (25)	7 (29)	18 (26)		
Professional/graduate Sschool	10 (21)	4 (17)	13 (19)		
**Grew up in traditional community**					
Yes	10 (17)	2 (8)	12 (17)		
No	30 (63)	19 (79)	49 (68)		
Some	8 (11)	3 (13)	11 (15)		
**Relocated from home community**					
Yes	25 (52)	10 (42)	35 (49)		
No	18 (38)	11 (46)	29 (40)		
N/A	5 (10)	3 (12)	8 (11)		
**Job permanence**					
Permanent	27 (56)	14 (58)	41 (57)		
Not permanent	17 (36)	9 (38)	26 (36)		
Other	4 (8)	1 (4)	5 (7)		
**Blood pressure medication**					
Yes	1 (2)	5 (21)	6 (8)		
No	32 (67)	18 (75)	50 (70)		
Missing	15 (31)	1 (4)	16 (22)		
**Cholesterol medication**					
Yes	1 (2)	2 (8)	3 (4)		
No	32 (67)	21 (88)	53 (74)		
Missing	15 (31)	1 (4)	16 (22)		
**Diabetes medication**					
Yes	1 (2)	1 (4)	2 (3)		
No	32 (67)	22 (92)	52 (72)		
Missing	15 (31)	1 (4)	16 (22)		
Indoor work	13.54 ± 9.29	12.78 ± 4.97	13.39 ± 8.00	1 (1.4)	0.72
Outdoor work	3.02 ± 3.18	6.17 ± 3.33	4.13 ± 3.50	1 (1.4)	**0.01**
Self-efficacy	32.30 ± 4.82	33.92 ± 3.40	32.86 ± 4.37	1 (1.4)	0.15
Métis identity	33.63 ± 5.84	34.08 ± 3.83	33.76 ± 5.19	1 (1.4)	0.70
SBP, mm Hg	116 ± 14	121 ± 12	118 ± 13	0	0.11
DBP, mm Hg	72 ± 8	76 ± 9	73 ± 9	0	0.07
HR, bpm	70 ± 11	62 ± 11	67 ± 11	0	**0.01**
cPWV, m/s	8.69 ± 3.87	8.80 ± 3.42	8.73 ± 3.70	4 (5.6)	0.91
upPWV, m/s	20.77 ± 10.64	22.46 ± 12.61	21.33 ± 11.27	6 (8.3)	0.90
lpPWV, m/s	9.61 ± 2.49	10.29 ± 2.32	9.83 ± 2.44	7 (9.7)	0.29
HRV-SDNN, ms	61.96 ± 47.66	80.30 ± 57.64	69.13 ± 51.95	1 (1.4)	0.16
HRV-RMSSD, ms	51.01 ± 38.31	79.90 ± 78.21	62.53 ± 58.04	1 (1.4)	0.10
HRV-LF, nu	36.7 ± 19.83	42.99 ± 19.56	38.35 ± 19.80	1 (1.4)	0.21
HRV-HF, nu	48.48 ± 20.81	40.89 ± 17.35	45.37 ± 19.84	1 (1.4)	0.13
HRV LF/HF, nu	1.10 ± 1.04	1.39 ± 1.14	1.19 ± 1.06	1 (1.4)	0.30
BRS-LF, nu	7.84 ± 5.11	9.88 ± 9.26	8.54 ± 6.82	8 (11.1)	0.26
All BRS, ms/mm Hg	15.20 ± 9.62	18.90 ± 15.08	16.45 ± 11.77	7 (9.7)	0.30

Values are n (%) or mean ± standard deviation, unless otherwise indicated. Sex coded as 0 = male, 1 = female. Boldface indicates significance.

bpm, beats per minute; BRS, baroreceptor sensitivity; c, central; DBP, diastolic blood pressure; HF, high frequency; HR, heart rate; HRV, heart rate variability; LF, low frequency; lp, lower-body peripheral; nu, normalized power units; PWV, pulse wave velocity; RMSDD, root mean square of successive differences; SBP, systolic blood pressure; SDNN, standard deviation of normal-to-normal intervals; up, upper-body peripheral. N/A, not available,

∗ *P*-values represent differences between male and female individuals, based on independent samples *t*-tests.

### Vascular measures—PWV

Table 2 summarizes significant findings for hierarchical models examining relationships of self-efficacy, gender roles (indoor and outdoor housework) and Métis identity with vascular (PWV) health indicators. In examining the relationships of self-efficacy, outdoor housework, and Métis identity, with lpPWV lpPWV, covariates age, sex, SBP, and HR entered in step 1 did not significantly explain the variance in lpPWV. The addition of main effects of self-efficacy, outdoor housework responsibility, and Métis identity in step 2 significantly increased the variance explained, from 11% to 29% (ΔF = 5.40, *P* < 0.01). Self-efficacy (β = –0.34, *P* < 0.01) and Métis identity (β = 0.29, *P* =0 .01) were significant contributors in this step. In step 3, the interaction term self-efficacy x outdoor housework was added, and no change occurred in the variance explained, suggesting no moderation effect of self-efficacy and outdoor work with lpPWV. The results, however, suggested that high self-efficacy was associated with reduced lpPWV, whereas high Métis identity affiliation was associated with increased lpPWV.

**Table 2:** Hierarchical regression models for relationships of self-efficacy and gender roles with vascular indicators in Métis adults in Saskatchewan

Vascular measures hierarchical regression models									
Self-efficacy, Outdoor yard work, Métis identity and lpPWV					Self-efficacy, Indoor housework, Métis identity and lpPWV				
Predictor	β	β ∗	95% CI (LL, UL)	*P*	Predictor	β	β ∗	95% CI (LL, UL)	*P*
**Step 1**				0.09	**Step 1**				0.09
Age	–0.04	–0.01	–0.04, 0.03	0.78	Age	–0.04	–0.01	–0.04, 0.03	0.78
Sex	–0.01	–0.07	–1.32, 1.19	0.92	Sex	–0.01	–0.07	–1.32, 1.19	0.92
SBP	0.32	0.06	0.01, 0.10	**0.02**	SBP	0.32	0.06	0.01, 0.10	**0.02**
HR	–0.16	–0.03	–0.08, 0.02	0.22	HR	–0.16	–0.03	–0.08, 0.02	0.22
**Step 2**				**< 0.01**	**Step 2**				**< 0.01**
Age	0.01	0.00	–0.04, 0.04	0.96	Age	–0.04	–0.01	–0.04, 0.03	0.76
Sex	–0.16	–0.77	–2.09, 0.56	0.25	Sex	–0.76	–0.38	–1.56, 0.81	0.53
SBP	0.21	0.04	–0.01, 0.08	0.11	SBP	0.24	0.04	–0.00, 0.09	0.07
HR	–0.14	–0.03	–0.08, 0.02	0.25	HR	–0.16	–0.03	–0.08, 0.02	0.17
Efficacy	–0.34	–0.18	–0.30, –0.06	**<0.01**	Efficacy	–0.35	–0.19	–0.30, –0.08	**<0.01**
Outdoor	–0.15	–0.10	–0.27, 0.06	0.22	Indoor	–0.02	–0.01	0.07, 0.06	0.83
Identity	0.29	0.13	0.03, 0.23	**0.01**	Identity	0.29	0.13	0.03, 0.23	**0.01**
**Step 3**				0.68	**Step 3**				0.20
Age	0.00	0.00	–0.04, 0.04	0.98	Age	–0.02	–0.00	–0.04, 0.03	0.86
Sex	-0.16	–0.81	–2.16, 0.54	0.23	Sex	–0.05	–0.23	–1.43, 0.97	0.70
SBP	0.21	0.04	–0.01, 0.08	0.12	SBP	0.22	0.04	–0.01. 0.08	0.08
HR	-0.13	–0.03	–0.08, 0.02	0.27	HR	–0.15	–0.03	–0.08, 0.02	0.19
Efficacy	-0.33	–0.18	–0.30, –0.06	**<0.01**	Efficacy	–0.27	–0.14	–0.28, –2.14	**0.04**
Outdoor	-0.15	–0.10	–0.27, 0.06	0.22	Indoor	–0.07	–0.02	–0.09, 0.05	0.54
Identity	0.28	0.13	0.03, 0.23	**0.01**	Identity	0.28	0.13	0.03, 0.23	**0.01**
Efficacy x outdoor	0.05	0.01	–0.03, 0.04	0.68	Efficacy x indoor	–0.16	–0.01	–0.03, 0.01	0.20

Sex coded as 0 = male, 1 = female. β indicates standardized Beta; β ∗ indicates unstandardized Beta. Boldface indicates significance.

CI, confidence interval; HR, heart rate; LL, lower limit; lpPWV, lower-body peripheral pulse wave velocity; SBP, systolic blood pressure; UL, upper limit.

Replacing outdoor housework with indoor work in the models yielded similar results for the relationship with lpPWV. A significant main effect explained 28% of the variance in lpPWV (ΔR^2^ = 0.17, ΔF = 4.79, *P* < 0.01). Gender roles were not related to lpPWV, though self-efficacy (β = –0.35, *P*<0.01) and Métis identity (β = 0.29, *P* = 0.01) were. The regression models examining the relationships with central and upper-body peripheral PWV were not significant.

### Neurovascular measures—baroreceptor sensitivity and HR variability

**Figure 1:**
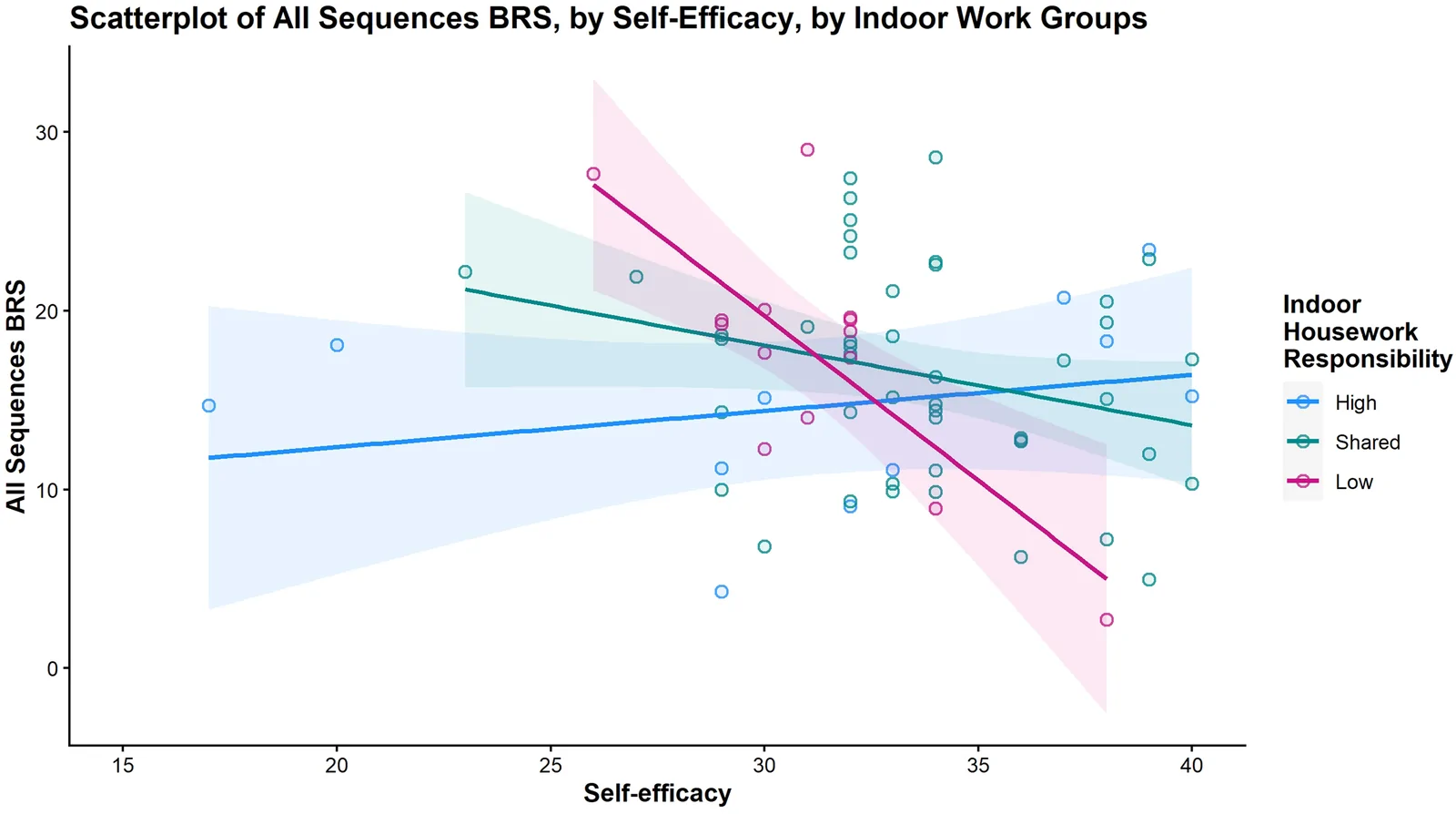
Simple slopes analysis of the significant interaction between self-efficacy and indoor housework on all-Sequence baroreceptor sensitivity (BRS) with 95% confidence intervals. Low: scores < –1 standard deviation (SD); Shared: scores > –1 SD and < 1 SD; High: scores > 1 SD.

Table 3 summarizes significant findings for hierarchical models examining relationships of self-efficacy, Métis identity, and gender roles with neurovascular (BRS and HRV) health indicators. Examining relationships of self-efficacy, indoor housework, and Métis identity, with all-sequence BRS, and covariates age, sex, SBP, and HR entered in step 1 significantly explained 20% of the variance in all-sequence BRS (ΔF = 4.10, *P* = 0.01). The addition of main effects of self-efficacy, indoor housework responsibility, and Métis identity in step 2 did not significantly increase the variance explained. In step 3, however, the added interaction term self-efficacy x indoor housework significantly increased the explained variance to 29%, suggesting a moderation effect of self-efficacy and indoor work with all-sequence BRS (ΔR^2^ = 0.05, ΔF = 4.80, *P* = 0.03). Covariates SBP (β = –0.26, *P* < 0.05) and HR (β = –0.27, *P* = 0.02), self-efficacy (β = –0.80, *P* = 0.02), and the interaction term self-efficacy x indoor work (β = 0.08, *P* = 0.03) were significant contributors in the model. Simple slopes analysis (Fig. 1) of the interaction indicated that the association between self-efficacy and all-sequence BRS differed based on level of indoor work responsibility. For individuals with low levels of indoor housework responsibility, higher self-efficacy was associated with lower levels of all-sequence BRS. The association of self-efficacy with all-sequence BRS was weaker for persons with shared indoor housework responsibility. No relationship was observed between all-sequence BRS and self-efficacy for persons with high levels of indoor housework responsibility.

**Table 3:** Hierarchical regression models for relationships of self-efficacy and gender roles with neurovascular indicators in Métis adults in Saskatchewan

Neurovascular measures hierarchical regression models									
Self-efficacy, Indoor housework, Métis identity and all BRS					Self-efficacy, Outdoor housework, Métis identity and HRV-HF				
Predictor	β	β∗	95% CI (LL, UL)	*P*	Predictor	β	β∗	95% CI (LL, UL)	*P*
**Step 1**				**0.01**	**Step 1**				0.24
Age	–0.14	–0.10	–0.27, 0.07	0.25	Age	–0.21	–0.26	–0.58, 0.07	0.09
Sex	–0.10	–2.47	–8.21, 3.27	0.39	Sex	0.26	10.81	–0.04, 21.66	0.05
SBP	–0.24	–0.20	–0.42, 0.02	0.07	SBP	0.11	0.16	–0.24, 0.57	0.43
HR	–0.25	–0.25	–0.49, –0.01	**0.04**	HR	–0.15	–0.26	–0.71, 0.19	0.25
**Step 2**				0.34	**Step 2**				0.96
Age	–0.15	–0.10	–0.28, 0.07	0.24	Age	–0.21	–0.26	–0.60, 0.09	0.14
Sex	–0.14	–3.26	–9.09, 2.58	0.27	Sex	0.25	10.53	–2.26, 23.32	0.11
SBP	–0.28	–0.24	–0.46, –0.02	**0.03**	SBP	0.10	0.14	–0.29, 0.57	0.51
HR	–0.26	–0.25	–0.49, –0.02	**0.04**	HR	–0.15	–0.26	–0.73, 0.20	0.27
Efficacy	–0.18	–0.46	–1.04, 0.12	0.12	Efficacy	–0.04	–0.18	–1.31, 0.95	0.76
Indoor	0.02	0.03	–0.29, 0.34	0.87	Outdoor	0.00	–0.01	–1.59, 1.58	0.99
Identity	0.14	0.31	–0.18, 0.79	0.21	Identity	0.06	0.26	–0.71, 1.17	0.63
**Step 3**				**0.03**	**Step 3**				**0.04**
Age	–0.18	–0.12	–0.29, 0.05	0.16	Age	–0.16	–0.19	–0.54, 0.20	0.26
Sex	–0.19	–4.44	–10.22, 1.34	0.13	Sex	0.30	12.43	–0.19, 25.05	0.05
SBP	–0.26	–0.22	–0.43, –0.01	**< 0.05**	SBP	0.09	0.14	–0.28. 0.56	0.51
HR	–0.27	–0.27	–0.50, –0.04	**0.02**	HR	–0.18	–0.31	–0.76, 0.15	0.19
Efficacy	–0.31	–0.80	–1.45, –0.16	**0.02**	Efficacy	–0.07	–0.30	–1.41, 0.81	0.59
Indoor	0.10	0.14	–0.18, –0.47	0.39	Outdoor	–0.01	–0.06	–1.61, 1.49	0.94
Identity	0.15	0.32	–0.16, 0.79	0.18	Identity	0.10	0.38	–0.55, 1.31	0.42
Efficacy x indoor	0.28	0.08	0.01, 0.16	**0.03**	Efficacy x outdoor	–0.26	–0.35	–0.68, 0.01	**0.04**

Sex coded as 0 = male, 1 = female. β indicates standardized Beta; β ∗ indicates unstandardized Beta. Boldface indicates significance.

BRS, baroreceptor sensitivity; CI, confidence interval; HF, high frequency; HR, heart rate; HRV, heart rate variability; LL, lower limit; SBP, systolic blood pressure; UL, upper limit.

**Figure 2:**
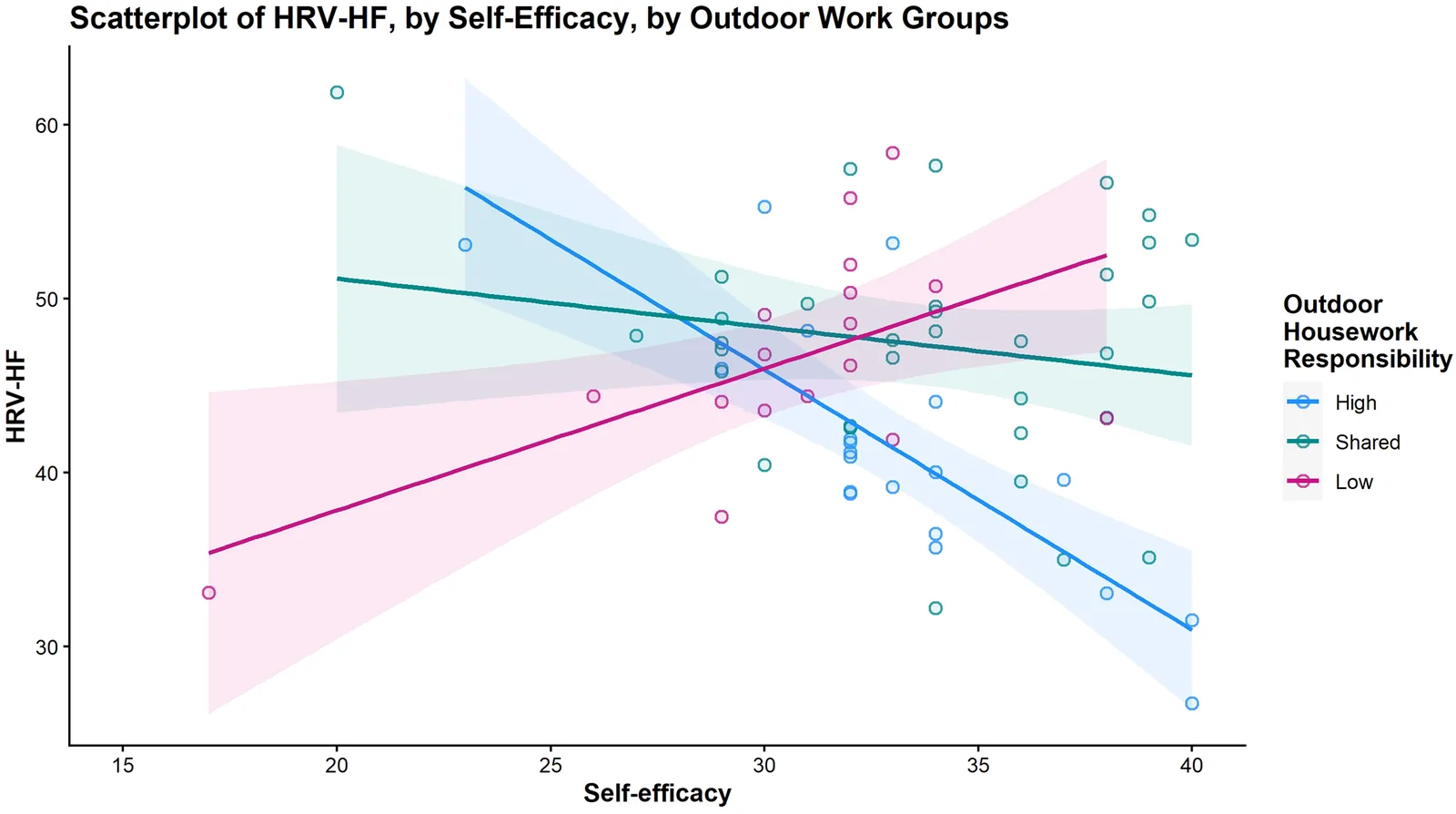
Simple slopes analysis of the significant interaction between self-efficacy and outdoor housework on heart rate variability–high frequency (HRV-HF) with 95% confidence intervals. Low: scores < –1 standard deviation (SD); shared: scores > –1 SD and < 1 SD; high: scores > 1 SD. BRS, baroreceptor sensitivity.

Examining relationships of self-efficacy, outdoor housework responsibility, and Métis identity, with HRV-high frequency(HF), and the covariates age, sex, SBP, and HR entered in step 1 did not significantly explain the variance in spectral HRV-HF. The addition of main effects of self-efficacy, outdoor housework responsibility, and Métis identity in step 2 also did not significantly increase the variance explained. However, in step 3, the added interaction term self-efficacy x outdoor housework significantly changed the explained variance to 14%, suggesting a moderation effect of self-efficacy and outdoor work with HRV-HF (ΔR^2^ = 0.06, ΔF = 4.21, *P* = 0.04). The interaction term self-efficacy x outdoor housework was a significant contributor to the model (β = –0.35, *P* = 0.04). Simple slopes analysis (Fig. 2) of the interaction indicated that the association between self-efficacy and HRV-HF differed based on level of outdoor housework responsibility. For individuals with high levels of outdoor housework responsibility, higher self-efficacy was associated with lower levels of HRV-HF. For individuals with low levels of outdoor housework responsibility, higher self-efficacy was associated with higher HRV-HF scores. No relationship was found between self-efficacy and HRV-HF for persons with shared outdoor housework responsibility. There was no significant finding for the models examining relationships with HRV–standard deviation of normal-to-normal intervals, HRV–root mean square of successive differences, HRV-low frequency/high frequency, and BRS-low frequency.

## Discussion

This research found that self-efficacy, gender roles, and Métis identity are significantly associated with parameters of vascular and neurovascular health in Métis people. Further, this study is the only known study to examine determinants of vascular and neurovascular health in Métis people, particularly using objective health assessment measures. This research adds to the understanding of CV health in relation to gender beyond binary gender-identity classifications. Our research also represents a unique perspective on how self-efficacy may relate to vascular and neurovascular health within the context of gendered division of labour.

A significant finding of this research is that Métis identity affiliation is associated with arterial stiffness in Métis people. Specifically, higher Métis identity affiliation was associated with stiffer lower-body peripheral arteries, suggesting poorer CV health. Although Métis identity is often discussed as a source of resilience and cultural strength,^36^, ^37^, ^38^ our findings suggest that the health benefits of cultural identity are not uniform for Métis people. This may be because Métis persons with stronger identity affiliation may experience increased discrimination, structural inequalities, or have a greater emotional burden of cultural responsibility, factors established by previous research as increasing chronic stress and by extension arterial stiffness.^39^^,^^40^ Previous research including other racialized groups indicates that stress related to everyday experiences of discrimination is associated with increased arterial stiffness.^41^ Although identity may have a positive association with health, including through kinship ties, community belonging, and self-determination,^15^^,^^36^^,^^42^ our findings suggest that for Métis people, cultural identity may also be linked to trauma and negative health experiences, which in turn negatively impacts CV health. The contrasting findings observed for lpPWV relative to the other PWV measures, however, may reflect important regional differences in vascular physiology, as lower-body peripheral arterial stiffness may be influenced by distinct behavioural, occupational, or circulatory factors relative to central vascular measures. Unlike central arteries, which are highly influenced by age-related elastin degradation and cumulative CV risk exposure, peripheral muscular arteries contain a greater proportion of vascular smooth muscle and demonstrate different stiffness characteristics and adaptive responses.^43^ The implication of our finding is that psychosocial stressors, whether based on historic or contemporary experiences, may have a more pervasive role in the peripheral vascular health of Métis people. Therefore, enhancing the health of Métis people requires an approach that is trauma-informed and culture-specific.

Self-efficacy was found to be significantly associated with PWV in Métis people. Higher self-efficacy was associated with lower peripheral PWV, suggesting reduced risk of CVD.^20^^,^^21^ This finding, however, differed from the positive association observed in the bivariate analyses, suggesting that shared variance among the psychosocial and demographic variables or potential confounding effects may have influenced the unadjusted relationship. The findings for these associations build on previous evidence with the general population, suggesting that perceived agency and control are associated with vascular and neurovascular pathways in Métis people through mechanisms such as reduced sympathetic activation and lower allostatic load.^16^^,^^22^^,^^44^ Our findings also align with experiences among Black women in the US, among whom low environment mastery is associated with greater PWV.^45^

Although our PWV findings support previous research involving Indigenous peoples indicating that self-efficacy is generally health enhancing,^14^ our study also uniquely identified that for Métis people, the physiological benefit of self-efficacy is highly contextual. For example, the association of self-efficacy with HRV-HF in Métis people depended on gender role. Participants with higher levels of self-efficacy and higher level of responsibility for outdoor housework had lower HRV-HF than did persons with high level of self-efficacy but low level of outdoor work responsibility, suggesting greater CVD risks in the former. Similarly, Métis persons with higher levels of self-efficacy had lower BRS when level of responsibility for indoor housework was low, also suggesting poorer CV health. This finding builds on previous research indicating that lower BRS in Indigenous peoples was associated with higher SBP, an association shaped by psychosocial factors, including physical activity and cultural identity.^18^ Our findings suggest that in Métis people, a high level of self-efficacy does not guarantee better CV autonomic regulation and depends on how gender roles are structured.

Household chores can be stress-inducing when they are associated with expectations, responsibility, and unequal division of labour.^46^ Given well established correlates of stress with HRV,^16^^,^^39^ our findings extend this evidence, showing a direct association between outdoor housework responsibility level and neurovascular health. Additionally, a high level of self-efficacy to perform outdoor chores may represent an increase in workload effect including metabolic equivalent of tasks, as persons may push themselves harder, take on more tasks, or engage in more physically demanding tasks. Given that in our research male individuals had higher levels of responsibility for outdoor tasks than female individuals, whereas indoor housework was shared equally, a high level of self-efficacy may suggest more internalized responsibility, overwork, or increased burden. Men may also experience more pressure to have competence in outdoor tasks or maintain traditional gendered expectations. Previous research has shown that self-efficacy is associated with participation in gendered outdoor activities such as hunting.^47^ These findings suggest that confidence in outdoor tasks is shaped by socially reinforced gender norms, increasing pressure to meet these expectations. These implications for health promotion are significant; initiatives should consider family roles and responsibilities modifiable determinants of CV health, emphasizing equitable sharing of household tasks to enhance health-promoting benefits.

Strengths of this research included focusing specifically on an underrepresented population and using culture-specific assessment measures. Given established associations between aging and vascular and neurovascular measures and the relatively young age of our sample (39 ± 16 years), a larger sample size with more age variations would facilitate age-stratified analyses, contributing to more comprehensive understandings of determinants of Métis people’s vascular and neurovascular health. In addition, although our research was sufficiently powered to detect differences, future larger samples will facilitate examining other interaction terms in the models, such as Métis identity x self-efficacy, or Métis identity x housework (indoor and outdoor), to explore moderating effects with these determinants. The number of regression analyses conducted in this exploratory study also also increases the possibility of type I error. Findings therefore should be interpreted cautiously and should be confirmed in larger studies. The novel exploration of self-efficacy and its association with gender roles in vascular and neurovascular health requires further investigation to gain comprehensive understandings of these associations. This study focused on gender roles as a key gendered construct; however, other gender-related parameters, such as income, employment status, education, caregiving burden, and access to material resources, may also be relevant and should be explored, to illuminate how gendered experiences shape health outcomes.

Additionally, except for the Métis identity scale, this study relied on measures that were initially designed for general populations. Subsequently, these measures may not fully reflect cultural specificity for Métis people, emphasizing the need for Métis-specific assessment tools. Further studies including the use of qualitative and culture-specific methods, such as storytelling, are needed to gain an in-depth understanding of these relationships.

## Conclusion

Our study found complex interactions between self-efficacy and gender roles, indicating that psychosocial factors, including self-efficacy, cultural identity, and gender roles, are associated with vascular and neurovascular health in Métis people. Persons with high levels of both self-efficacy and responsibility for outdoor housework had poorer HRV. Low levels of responsibility for indoor housework and high levels of self-efficacy were associated with lower BRS sequences, indicating poorer autonomic functioning and CV health. Vascular and neurovascular health may be enhanced by developing community-driven strength-based approaches, grounded in Métis peoples’ resilience, self-determination, and cultural practices.

## Ethics Statement

The research reported in this paper adhered to the Strengthening the Reporting of Observational Studies in Epidemiology (STROBE) Statement for cross-sectional studies guidelines.

## Patient Consent

The authors confirm that an informed participant consent form has been obtained for this article.

## Funding Sources

H.F. received funding from Heart and Stroke/Canadian Institutes of Health Research Early Career Indigenous Women’s Heart and Brain Health Chair (Grant # WH1-160082).

## Disclosures

A.M. reports being a former consultant with Métis Nation-Saskatchewan and a current executive member of Saskatoon Métis Local 126.

## Footnotes

To access the supplementary material accompanying this article, visit *CJC Open* at https://www.cjcopen.ca/ and at https://doi.org/10.1016/j.cjco.2026.05.015

## References

[1] Statistics Canada Indigenous population continues to grow and is much younger than the non-Indigenous population, although the pace of growth has slowed. https://www150.statcan.gc.ca/n1/daily-quotidien/220921/dq220921a-eng.htm.

[2] Macdougall B. Land, family and identity: contextualizing Metis health and well-being. https://www.ccnsa-nccah.ca/docs/context/RPT-ContextualizingMetisHealth-Macdougall-EN.pdf.

[3] Atzema C.L., Khan S., Lu H. (2015). Cardiovascular disease rates, outcomes, and quality of care in Ontario Métis: a population-based cohort study. PLoS One.

[4] Randall J., Svenson L., Maximova K. (2019). The burden of hypertension and heart disease amongst the Métis Nation of Alberta.

[5] Ralph-Campbell K., Oster R.T., Connor T. (2009). Increasing rates of diabetes and cardiovascular risk in Métis settlements in northern Alberta. Int J Circumpolar Health.

[6] Abate K.H., Abebe Z., Abera S.F. (2018). Global, regional, and national age-sex-specific mortality for 282 causes of death in 195 countries and territories, 1980–2017: a systematic analysis for the Global Burden of Disease Study 2017. Lancet.

[7] Ammirati E., Barengo N.C., Beaton A. (2020). Global burden of cardiovascular diseases and risk factors, 1990-2019: update from the GBD 2019 Study. J Am Coll Cardiol.

[8] Humphries K.H., Izadnegahdar M., Sedlak T. (2017). Sex differences in cardiovascular disease—impact on care and outcomes. Front Neuroendocrinol.

[9] Powell-Wiley T.M., Baumer Y., Baah F.O. (2022). Social determinants of cardiovascular disease. Circ Res.

[10] Schwartz S.J., Zamboanga B.L., Weisskirch R.S. (2008). Broadening the study of the self: integrating the study of personal identity and cultural identity. Soc Personal Psychol Compass.

[11] Thoits P.A. (2011). Mechanisms linking social ties and support to physical and mental health. J Health Soc Behav.

[12] Eisenberger N.I., Cole S.W. (2012). Social neuroscience and health: neurophysiological mechanisms linking social ties with physical health. Nat Neurosci.

[13] Sweet S.N., Fortier M.S., Strachan S.M., Blanchard C.M. (2012). Testing and integrating self-determination theory and self-efficacy theory in a physical activity context. Can Psychol.

[14] Klassen G.A.H., Cole D., Klassen R. (2024). An exploration of self-continuity for rural Indigenous youth: considering the influence of community and cultural factors on perceiving oneself across time. Transcult Psychiatry.

[15] Foulds H.J.A., LaFleur J., Johnson S.R. (2025). “Community traditions, community kinship, language, and land bring me a lot of joy”: the importance of culture and social support in the health and wellbeing of Métis people. Int J Qual Stud Health Well-being.

[16] Kim H.G., Cheon E.J., Bai D.S., Lee Y.H., Koo B.H. (2018). Stress and heart rate variability: a meta-analysis and review of the literature. Psychiatry Investig.

[17] Gentilin A., Cevese A., Schena F., Tarperi C. (2023). Mental stress augments central artery stiffness in young individuals of both sexes. Biol Psychol.

[18] Foulds H.J.A., Bredin S.S.D., Warburton D.E.R. (2018). Ethnic differences in vascular function and factors contributing to blood pressure. Can J Public Health.

[19] Foulds H.J.A., Bredin S.S.D., Warburton D.E.R. (2016). The vascular health status of a population of adult Canadian Indigenous peoples from British Columbia. J Hum Hypertens.

[20] Nilsson P.M., Khalili P., Franklin S.S. (2014). Blood pressure and pulse wave velocity as metrics for evaluating pathologic ageing of the cardiovascular system. Blood Press.

[21] Darvish S., Mahoney S.A., Venkatasubramanian R. (2024). Socioeconomic status as a potential mediator of arterial aging in marginalized ethnic and racial groups: current understandings and future directions. J Applied Physiol (1985).

[22] Mccraty R., Shaffer F. (2015). Heart rate variability: new perspectives on physiological mechanisms, assessment of self-regulatory capacity, and health risk. Glob Adv Health Med.

[23] Herzog M.J., Müller P., Lechner K. (2025). Arterial stiffness and vascular aging: mechanisms, prevention, and therapy. Signal Transduct Target Ther.

[24] Johnson S.R., Chilibeck P., Oosman S.N., Foulds H.J.A. (2024). A scoping review of Indigenous community-specific physical activity measures developed with and for Indigenous peoples in Canada, Australia, and New Zealand. Appl Physiol Nutrition Metab.

[25] Gaudry A. (2009). The Canadian encyclopedia.

[26] Statistics Canada (2021). Projections of the Indigenous populations and households in Canada, 2016 to 2041.

[27] Chen G., Gully S.M., Eden D. (2001). Validation of a new general self-efficacy scale. Organ Res Methods.

[28] Statistics Canada (2016). General social survey C31 main survey- family.

[29] Canadian Society for Exercise Physiology (2019).

[30] Díaz A., Galli C., Tringler M. (2014). Reference values of pulse wave velocity in healthy people from an urban and rural Argentinean population. Int J Hypertens.

[31] Laborde S., Mosley E., Thayer J.F. (2017). Heart rate variability and cardiac vagal tone in psychophysiological research – recommendations for experiment planning, data analysis, and data reporting. Front Psychol.

[32] Parati G., DiRienzo M., Mancia G. (2000). How to measure baroreflex sensitivity: from the cardiovascular laboratory to daily life. J Hypertens.

[33] O’Neil A., Scovelle A.J., Milner A.J., Kavanagh A. (2018). Gender/sex as a social determinant of cardiovascular risk. Circulation.

[34] Cohen J. (1988).

[35] Faul F., Erdfelder E., Buchner A., Lang A.G. (2009). Statistical power analyses using G power 3.1: tests for correlation and regression analyses. Behav Res Methods.

[36] Cameron R., Richer D., Bird M., Fuller-Thomson E. (2025). “I can stand tall and be a Métis person and just be proud of it”: pathways to a flourishing Métis identity. J Indig Soc Dev.

[37] Auger M.D. (2021). Understanding our past, reclaiming our culture: Métis resistance, resilience, and connection to land in the face of colonialism. J Indigenous Social Dev.

[38] Halseth R., Murdock L. Supporting Indigenous self-determination in health: lessons learned from a review of best practices in health governance in Canada and internationally. National Collaborating Centre for Indigenous Health. https://www.nccih.ca/Publications/Lists/Publications/Attachments/317/Ind-Self-Determine-Halseth-Murdoch-LC-2023-06-08-VS-EN-003-WEB.pdf.

[39] Logan J.G., Teachman B.A., Liu X. (2020). Acute psychological stress, autonomic function, and arterial stiffness among women. Int J Psychophysiol.

[40] Suglia S.F., Shelton R.C., Factor-Litvak P. (2025). Stress across the lifecourse and adult mental and physical health outcomes. Ann Behav Med.

[41] Bromfield S.G., Sullivan S., Saelee R. (2020). Race and gender differences in the association between experiences of everyday discrimination and arterial stiffness among patients with coronary heart disease. Ann Behav Med.

[42] Gabel C., Henry R., Nychuk A., Hartmann S., LaVallee A. (2024). “We know who we are”: reflections on Métis youth identity, health and well-being. Int J Indig Health.

[43] Bentley G.J., Barrell A.M., McEniery C.M. (2025). Central and peripheral artery stiffness: key players in cardiovascular disease risk. J Cardiovasc Comput Tomogr.

[44] Pilz N., Heinz V., Ax T. (2024). Pulse wave velocity: methodology, clinical applications, and interplay with heart rate variability. Rev Cardiovasc Med.

[45] Blevins K.M., Fields N.D., Pressman S.D. (2025). Superwoman schema and arterial stiffness in Black American women: assessing the role of environmental mastery. Ann Behav Med.

[46] Ervin J., Taouk Y., Alfonzo L.F., Hewitt B., King T. (2022). Gender differences in the association between unpaid labour and mental health in employed adults: a systematic review. Lancet Public Health.

[47] Smith A.P., Metcalf E.C., Nesbitt H.K. (2022). Confidence, community & conservation: exploring the relationship between self-efficacy and experience in female hunters. J Outdoor Rec Tourism.

