# Supplementary material for: Perceived Self-Efficacy, Gender Roles, and Cultural Identity As Determinants of Vascular and Neurovascular Health in Métis People in Canada
Source: CJC Open. 2026 Jun 4;8(9):1188–97. doi: 10.1016/j.cjco.2026.05.015 (PMC13583569; doi:10.1016/j.cjco.2026.05.015)
Supplement: Supplementary file 1 [file mmc1.docx]

Supplemental Table S1*.* Intercorrelations Among Cultural Identity, Gender Roles, Vascular and Neurovascular Variables of Métis Adults in Saskatchewan

| Variable | Age | SBP | HR | cPWV | uPWV | lpPWV | HRV-SDNN | HRV-RMSDD | HRV-HF | HRV-LF/HF | BRS-LF | ALL BRS | In-door | Out-door | ID | Effi |
| --- | --- | --- | --- | --- | --- | --- | --- | --- | --- | --- | --- | --- | --- | --- | --- | --- |
| Age | 1 |  |  |  |  |  |  |  |  |  |  |  |  |  |  |  |
| SBP | **0.43*** | 1 |  |  |  |  |  |  |  |  |  |  |  |  |  |  |
| HR | -0.07 | 0.11 | 1 |  |  |  |  |  |  |  |  |  |  |  |  |  |
| cPWV | **0.36*** | **0.26*** | 0.07 | 1 |  |  |  |  |  |  |  |  |  |  |  |  |
| uPWV | **0.28*** | 0.12 | -0.05 | **0.87*** | 1 |  |  |  |  |  |  |  |  |  |  |  |
| lpPWV | 0.13 | **0.30*** | -0.13 | 0.06 | 0.16 | 1 |  |  |  |  |  |  |  |  |  |  |
| HRV-SDNN | -0.03 | -0.06 | -0.22 | 0.15 | -0.05 | -0.16 | 1 |  |  |  |  |  |  |  |  |  |
| HRV-RMSDD | -0.09 | -0.13 | -0.21 | **-0.25*** | **-0.28*** | -0.04 | **0.78*** | 1 |  |  |  |  |  |  |  |  |
| HRV-HF | -0.15 | -0.05 | -0.04 | -0.11 | -0.1 | 0.16 | -0.02 | 0.05 | 1 |  |  |  |  |  |  |  |
| HRV- LF/HF | -0.02 | -0.04 | -0.10 | 0.01 | -0.06 | -0.16 | -0.17 | **-0.24*** | **-0.75*** | 1 |  |  |  |  |  |  |
| BRS-LF | -0.03 | -0.19 | **-0.39*** | **-0.40*** | **0.35*** | -0.09 | **-0.48*** | **0.56*** | 0.01 | 0.04 | 1 |  |  |  |  |  |
| All BRS | **-0.27*** | **-0.32*** | **-0.32*** | **-0.35*** | **0.33*** | -0.08 | **0.47*** | **0.63*** | 0.21 | -0.14 | **0.45*** | 1 |  |  |  |  |
| Indoor | 0.21 | 0.12 | 0.06 | 0.13 | 0.11 | -0.05 | 0.06 | 0.02 | -0.01 | -0.15 | 0.02 | -0.09 | 1 |  |  |  |
| Outdoor | 0.19 | 0.05 | -0.03 | 0.05 | 0.06 | -0.12 | 0.12 | 0.21 | -0.14 | 0.01 | 0.19 | 0.12 | **0.49*** | 1 |  |  |
| ID | 0.08 | 0.16 | 0.07 | 0.15 | 0.15 | **0.26*** | 0.12 | 0.10 | 0.03 | -0.10 | 0.07 | 0.05 | -0.05 | 0.04 | 1 |  |
| Effi | -0.07 | -0.09 | -0.04 | 0.05 | 0.13 | **0.31*** | 0.00 | -0.11 | -0.06 | -0.01 | -0.03 | -0.09 | 0.05 | 0.16 | 0.16 | 1 |

Notes: BRS- Baroreceptor Sensitivity; cPWV - Central Pulse Wave Velocity; Effi- Self-Efficacy; HF- High Frequency; HR-Heart Rate; HRV – Heart Rate Variability; ID- Métis Identity; Indoor- Indoor Housework Responsibility; LF- Low Frequency; lpPWV – Lower Peripheral Pulse Wave Velocity; Outdoor- Outdoor Housework Responsibility; RMSDD- root mean square of successive differences; SBP- Systolic Blood Pressure; SDNN - standard deviation of normal-to-normal intervals; uPWV- Upper Pulse Wave Velocity; *p<0.05.
